# The Interplay Between Systemic Inflammatory Factors and MicroRNAs in Age-Related Macular Degeneration

**DOI:** 10.3389/fnagi.2019.00286

**Published:** 2019-10-22

**Authors:** Zofia Litwińska, Anna Sobuś, Karolina Łuczkowska, Aleksandra Grabowicz, Katarzyna Mozolewska-Piotrowska, Krzysztof Safranow, Miłosz Piotr Kawa, Bogusław Machaliński, Anna Machalińska

**Affiliations:** ^1^Department of General Pathology, Pomeranian Medical University, Szczecin, Poland; ^2^First Department of Ophthalmology, Pomeranian Medical University, Szczecin, Poland; ^3^Department of Biochemistry and Medical Chemistry, Pomeranian Medical University, Szczecin, Poland

**Keywords:** macular degeneration, miRNA, cytokines, interleukin, inflammation

## Abstract

We aimed to explore the expression of systemic inflammatory factors and selected intracellular miRNAs that regulate inflammatory signaling pathways potentially involved in age-related macular degeneration (AMD) pathogenesis. A total of 179 patients with wet AMD, 175 with dry AMD and 121 controls were enrolled in the study. Soluble inflammatory factors were analyzed in plasma samples using Luminex technology. Expression of selected miRNAs was analyzed in isolated nucleated peripheral blood cells (PBNCs) using real-time qPCR. Wet AMD was an independent factor associated with higher concentrations of IL-6 (β = +0.24, *p* = 0.0004), GM-CSF (β = +0.31, *p* < 0.001), IFN-γ (β = +0.58, *p* < 0.001), higher expression of miRNA-23a-3p (β = +0.60, *p* < 0.0001), miRNA-30b (β = +0.32, *p* < 0.0001), miRNA-191-5p (β = +0.28, *p* < 0.0001) and lower concentration of IL-1β (β = −0.25, *p* = 0.0003), IL-5 (β = −0.45, *p* < 0.001), IL-10 (β = −0.45, *p* < 0.001), IL-12 (β = −0.35, *p* < 0.001), lower expression of miRNA-16-5p (β = −0.31, *p* < 0.0001), miRNA-17-3p (β = −0.18, *p* = 0.01), miRNA-150-5p (β = −0.18, *p* = 0.01) and miRNA-155-5p (β = −0.47, *p* < 0.0001). Multivariate analysis revealed that dry AMD was an independent factor associated with higher concentration of GM-CSF (β = +0.34, *p* < 0.001), IL-6 (β = +0.13, *p* = 0.05), higher expression of miRNA-23a-3p (β = +0.60, *p* < 0.0001), miRNA-126-3p (β = +0.23, *p* = 0.0005), miRNA-126-5p (β = +0.16, *p* = 0.01), miRNA 146a (β = +0.14, *p* = 0.03), and mRNA191-5p (β = +0.15, *p* = 0.03) and lower concentrations of TNF-α (β = +0.24, *p* = 0.0004), IL-1β (β = −0.39, *p* < 0.001), IL-2 (β = −0.20, *p* = 0.003), IL-5 (β = −0.54, *p* < 0.001), IL-10 (β = −0.56, *p* < 0.001), IL-12 (β = −0.51, *p* < 0.001), lower expression of miRNA-16-5p (β = −0.23, *p* = 0.0004), miRNA-17-3p (β = −0.20, *p* = 0.003) and miRNA-17-5p (β = −0.19, *p* = 0.004). Negative correlations between visual acuity and WBC, lymphocyte count, TNF-α, IL-1 β, IL-2, IL-4, IL-6, IL-10 concentrations and miRNA-191-5p, as well as positive correlations between visual acuity and miRNA-126-3p, -126-5p, and -155-5p PBNCs expression were found in AMD patients. No such correlations were found in the control group. Our results may suggest the role of both intra- and extracellular mechanisms implicated in inflammatory response regulation in multifactorial AMD pathogenesis.

## Introduction

Age-related macular degeneration (AMD) is a progressive and degenerative eye disease that is a leading cause of vision loss among the elderly population (Hernandez-Zimbron et al., 2018). It is estimated that the number of individuals affected by AMD in 2020 will reach 196 million and that the number will grow to 288 million in 2040 (Wong et al., 2014). Because of its socioeconomic impact together with the growing incidence and severity of the disease, AMD has become a major challenge in ophthalmology in recent years. However, the exact pathophysiology of AMD has not been fully elucidated, which hampers the possibility of developing efficient therapeutic options.

Two major clinical subtypes of AMD have been established: dry (atrophic) and wet (exudative, neovascular) AMD. Dry AMD is characterized by drusen accumulation and progressive geographic atrophy of the retinal pigment epithelium (RPE) and retina, whereas wet AMD is described by the growth of largely malformed leaky choroidal vessels into the retina (Feehan et al., 2011). The prevalence of dry AMD is higher than that of wet AMD, comprising 80–85% of all cases; however, wet AMD accounts for 90% of all clinical cases with loss of sight, and the rapid visual acuity loss in wet AMD may occur within several months (Ayoub and Patel, 2009). The pathogenesis of AMD remains unclear, although both genetic and environmental factors play a role. The complex etiology of this disease has been linked to various cellular, biochemical, and molecular events, from which inflammation emerges as crucial in AMD pathogenesis and progression (Nowak, 2006; Kauppinen et al., 2016).

Inflammation is a complex response induced by foreign material and/or tissue damage and is highly advantageous in the short term because it eliminates dangerous stimuli and initiates tissue recovery. However, long-term inflammation is detrimental; prolonged low-level inflammation has been linked with chronic conditions, such as cancer, diabetes, neurodegenerative diseases and others (Hunter, 2012).

The first report of inflammatory processes involved in AMD histopathology was published in Hegner (1916). Since then, several research groups have established that aggregates of choroidal leukocyte infiltrates are associated with disciform macular lesions (Green and Key, 1977). In further studies, Penfold et al. (1984) demonstrated the involvement of inflammatory cells (including macrophages, lymphocytes, and mast cells) in RPE atrophy and Bruch’s membrane breakdown. Macrophages release a range of mediators, mainly pro-inflammatory and pro-angiogenic, and their recruitment has been proposed in both wet and dry AMD forms (Ambati et al., 2013). Microglia, the immunocompetent resident macrophages, are equally distributed in the plexiform layers of the retina and possess a wide array of immune surface proteins (receptors for complement components, cytokines, chemokines) to sense their environment for on/off inflammatory signals (Karlstetter et al., 2014; Rashid et al., 2018). Microglial cells when activated in degenerative process may release different inflammatory mediators including cytokines, chemokines, reactive oxygen species (ROS), and nitric oxide (NO), all of them contributing to the maintenance of a chronic neuroinflammatory environment that initially plays essential role in protecting retinal tissue during the early steps of its degeneration (Cuenca et al., 2014). However, continuous presence of proinflammatory stimuli induces locally the cellular damage which together with retinal drusen can subsequently attract other reactive systemic immune cells to the retina (Arroba et al., 2018). In case of prolonged insult to the retina, the overreactive neurotoxic microglia release large amounts of pro-inflammatory and cytotoxic factors such as TNF-α and IL-1β, creating a proinflammatory environment favorable for further recruitment of retinal microglia and exogenous infiltrating monocytes (Scholz et al., 2015; Zhao et al., 2015). Recent findings of Natoli et al. (2018) confirm the interplay between local and systemic response in AMD: the group has shown that photo-oxidative damage induced an early pro-inflammatory and chemokine-driven response which triggered microglia/macrophage recruitment to the photoreceptor lesion. Although the clinical presentation of AMD is mainly limited to the retina, this notable involvement of inflammatory cells points to a possible systemic inflammatory response occurring with AMD. Since systemic activation of the complement cascade has also been observed in AMD patients (Machalinska et al., 2009; Machalinska et al., 2012; Kawa et al., 2014), it is possible that inflammatory factors in the circulation could also contribute to the pathogenesis of AMD via exchange between the choroid and the retina (Cao et al., 2013). Several studies have shown altered inflammatory cytokine profiles in AMD patients (Sato et al., 2018), highlighting their potential role in the pathogenesis of this disease.

Another evidence of circulating factors involvement in AMD pathogenesis comes from studies on drusen constituents – many of them are serum proteins (Johnson et al., 2011). The drusen are also composed of inflammatory factors and complement cascade components (Crabb, 2014). The accumulation of intracellular lipofuscin in the RPE triggers intracellular oxidative stress and subsequent inflammation by activation of innate immune cells of myeloid origin such as macrophages and microglia.

On the other hand in wet form of disease the injured RPE produces VEGF, interleukin-8 (IL-8) and monocyte chemoattractant protein-1 (MCP), which attract monocytes from the choriocapillaris along the outer surface of Bruch’s membrane (Lopez et al., 1996) resulting in the breakdown of the blood-retinal barrier and development of new vessels. The macrophages then express TNF-α and interleukin-1 (IL-1), which up-regulate complement factor-B (Wang et al., 2009), activate the complement alternative pathway in the subretinal space, and stimulate RPE cells to produce even more VEGF (Grossniklaus et al., 2002). Parallel to VEGF, the pro-inflammatory cytokine, interleukin 6 (IL-6) has also been found to play an important role in the development of CNV. IL-6 was found to be increased in the serum and eye aqueous humor of patients with neovascular AMD (Izumi-Nagai et al., 2007). Pongsachareonnont et al. (2018) reported a significant decrease in IL-6 levels due to regression of CNV that occurred in response to anti-VEGF treatment.

As AMD combines neovascularization with retinal inflammation, the suppression of inflammation and vessel growth prevention appears as the two emerging strategies for its treatment. Nowadays, the anti-inflammatory treatment is being widely investigated with a number of approaches, including complement inhibition (Damico et al., 2012). However, the results are still far from satisfactory.

A major role in governing various pathological processes that contribute to AMD pathogenesis, including inflammation, is attributed to microRNAs (miRNAs, miRs) (Kawa and Machalińska, 2014). These small, non-coding RNAs of approximately 20 nucleotides are potent gene expression regulators that have been found in a variety of body fluids, i.e., blood, saliva, and urine (Engels and Hutvagner, 2006). Changes in miRNA expression have been implicated in common human disorders such as cardiovascular, autoimmune and neurodegenerative diseases, or in inflammatory states. Subsequently, several studies have been performed to characterize the expression of various miRNAs both intracellularly and extracellularly, advocating the role of miRNAs as potential diagnostic and prognostic markers (Ardekani and Naeini, 2010). miRNAs act as “fine-tuners” of the immune system, regulating both pro- and anti-inflammatory actions (Dai and Ahmed, 2011). Their expression in stimulated immune or bystander cells can be altered, affecting inflammatory processes (Kroesen et al., 2015). In addition, intracellular miRNAs have been implicated as powerful endogenous factors in regulating inflammatory signaling cascades. Alternatively, as a part of the inflammatory response, the immune system is able to regulate the endogenous biogenesis of miRNAs at multiple levels (Bronevetsky and Ansel, 2013). Previously, “inflammatory” miRNA altered expression has been shown in retinal tissue and blood plasma (Berber et al., 2017; Pogue and Lukiw, 2018) as well as in peripheral blood mononuclear cells from AMD patients (Lin et al., 2018).

In the present study, we aimed to explore the expression of systemic inflammatory factors and selected intracellular miRNAs that regulate various inflammatory signaling pathways and processes potentially involved in AMD pathogenesis. We also focused on possible correlations of their expression with disease severity.

## Materials and Methods

### Characteristics of the Study Group

Three hundred fifty-four patients with newly diagnosed AMD recruited from the outpatient population of the First Department of Ophthalmology of Pomeranian Medical University in Szczecin, Poland, were included in the study. The control group consisted of 121 age-matched participants with no symptoms or signs of macular degeneration (defined as the absence of drusen, pigmentary abnormalities or neovascularization). All of the enrolled subjects underwent a complete ophthalmic examination, i.e., visual acuity and intraocular pressure measurements, optical coherence tomography (OCT) analysis (Spectralis, Heidelberg Engineering, Heidelberg, Germany) and dilated fundus examination using slit-lamp biomicroscopy. Visual acuity (VA) was measured using a Snellen letter chart and transformed to LogMAR (Logarithm of the minimum angle of resolution) for statistical analyses.

In the group of recruited AMD patients, 179 subjects had a clinical diagnosis of wet AMD, with newly diagnosed choroidal neovascularization (CNV) characterized by serous or hemorrhagic retinal pigment epithelium detachment, subretinal neovascular membrane, subretinal hemorrhage, or fibrous scar. The rest of the examined AMD patients, i.e., one hundred seventy-five subjects, had a clinical diagnosis of dry AMD, with visible alterations in RPE in the form of geographic retinal atrophy and macular drusen. In the case of different stages of the disease being diagnosed in each eye, the subject was categorized according to the severity of changes in the worse eye. Exclusion criteria included significant chronic systemic conditions, for example, collagen or neoplastic disease, diabetes mellitus, renal failure, hepatic dysfunction or any evidence of retinal disease except AMD (in AMD groups), i.e., glaucoma or intraocular inflammatory diseases.

Data regarding medical history, current drug use, working conditions and smoking status were collected based on laboratory data, pathology tests, and other information, with a particular focus on heart and vascular conditions and pre-existing arterial hypertension. Furthermore, the actual arterial BP was directly measured prior to ophthalmic examination in all subjects using a non-invasive BP system with a manual aneroid manometer. The mean result from three measurements obtained with 5-min resting intervals was calculated. From the obtained BP data, the systemic mean arterial pressure (MAP) was calculated as follows: MAP = diastolic BP + 1/3 (systolic BP − diastolic BP) mmHg. Furthermore, the following medical parameters were assessed in all patients: waist circumference [cm], waist/hip ratio (WHR), and body mass index (BMI) [weight (kg)/height (m)^2^]. Cumulative pack-years were calculated using the reported average number of cigarettes smoked per day and the number of years of smoking.

The study adhered to the tenets of the Declaration of Helsinki, and approval was obtained from the Local Research Ethics Committee. Moreover, each patient provided written informed consent for his or her involvement.

### Blood Sample Collection

Venous blood samples (∼7.5 ml) collected in EDTA tubes were centrifuged (2000 rpm, 4°C, 10 min), and the plasma was stored at −20 to −80°C until assayed. The red blood cells were lysed using BD Pharm Lyse lysing buffer (BD Biosciences, San Jose, CA, United States) for 15 min at room temperature to isolate peripheral blood nuclear cells (PBNCs).

### Luminex Assay

TNF-α, IL-2, IL-1b, IL-4, IL-5, IL-6 IL-8, IL-10, IL-12 p70, GM-CSF, and IFN-γ concentrations were measured in plasma by multiplex fluorescent bead-based immunoassays (Luminex Corporation, Austin, TX, United States) using commercial R&D Systems Luminex Performance Human High Sensitivity Cytokine Magnetic Panel A (R&D Systems, Minneapolis, MN, United States). A total of 100 μL of blanks, standards and samples were added to the plate together with a Microparticle Cocktail and incubated in the dark for 3 h at room temperature on a horizontal orbital microplate shaker set at 800 rpm. After this step, the wells were washed with 100 μL of wash buffer three times by using a hand-held magnet. Biotin-antibody cocktail (50 μL) was added to the plate and incubated with agitation at room temperature for 60 min in the dark. After washing, 50 μL of streptavidin–PE was added to each well and incubated in the dark for 30 min on a plate shaker. Finally, after washing, the microspheres in each well were resuspended in 100 μL wash buffer and shaken for 2 min at room temperature. The plate was read and analyzed on the Luminex 200 analyzer, and cytokine concentrations were determined from seven different standard curves showing median fluorescence intensity vs. protein concentration.

### miRNA Analysis

We chose 13 miRNAs involved in inflammatory signaling pathways and processes potentially contributing to AMD to be tested in this study (miRNA-16-5p, miRNA-17-3p, miRNA-17-5p, miRNA-21-3p, miRNA-23a-3p, miRNA-30b, miRNA-126-3p, miRNA-126-5p, miRNA-146a, miRNA-150-5p, miRNA-155-5p, miRNA-191-5p, miRNA-223-3p) (Dai and Ahmed, 2011; Kawa and Machalińska, 2014; Berber et al., 2017). miRNA for molecular analysis was obtained from PBNCs. Cellular RNA was isolated from 3 × 10^6^ nucleated cells using the mirVana Isolation Kit with organic phenol extraction (Thermo Fisher Scientific, Waltham, MA, United States) according to the manufacturer’s protocol. RNA concentration was measured using an Epoch Microplate Spectrophotometer (BioTek, Winooski, VT, United States), and 100 ng was used for first strand cDNA synthesis. For cDNA synthesis, 4 μL of each sample was used. First strand cDNA synthesis was performed in all samples using a qScript microRNA cDNA Synthesis Kit (Quantabio, Beverly, MA, United States). qPCR for the assessment of miRNA expression was performed with Bio-Rad CFX96 Real-Time Detection System (Bio-Rad, CA, United States). The reaction solution consisted of 1 μL of cDNA sample, iQ SYBR Green Supermix (Bio-Rad, CA, United States), Universal Primer provided in qScript microRNA Synthesis Kit and a forward primer specific to miRNA analysis. Quantification of the target miRNA expression value was expressed as 2^ΔCt^. To find the best reference gene, the NormFinder algorithm was used (Andersen et al., 2004); miR-93 was set as a reference miRNA.

### Statistical Analysis

Quantitative parameters measured in both eyes were averaged before further analysis. Since the distribution of the quantitative variables was significantly different from the normal distribution in most cases, the non-parametric Kruskal–Wallis test followed by Siegel and Castellan’s test was used to compare values between groups, and Spearman’s rank correlation coefficient (Rs) was used to measure the strength of associations between them. Fisher’s exact test was used to compare qualitative variables between groups. Multivariate analysis of AMD as an independent variable associated with concentrations of inflammatory factors and miRNAs or cell counts was performed using a general linear model (GLM) adjusted for age, sex and smoking status (pack-years), with logarithmic transformation applied to the dependent variables to normalize their distributions. Standardized regression coefficient (β) was used to measure strength of associations between independent and dependent variables. The sign of the Rs or β values (calculated for uni- and multivariate analysis, respectively) indicates the direction of the association (positive or negative), while higher absolute value (closer to −1 or +1) indicates stronger association. *P* < 0.05 was considered statistically significant. Statistica 13 software (Dell Inc., OK, United States) was used for statistical analysis.

## Results

### Characteristics of the Study Subjects

We enrolled 354 patients with AMD and 121 healthy controls in the study. A total of 175 patients presented with dry AMD and 179 with wet AMD. The clinical characteristics of the patients and controls are summarized in Table 1. Since epidemiological data collected so far indicate unquestionably that AMD is associated with the atherosclerosis we analyzed vascular-related risk factors in the study groups. The AMD and control groups were not significantly different as regards age and well-known atherosclerotic risk factors, including hypertension, history of ischemic heart disease, cardiac infarction, cerebral stroke, peripheral artery disease, and aortic aneurysm. The rate of past smokers and the number of smoking pack-years were significantly higher in wet than in dry AMD patients; these values were also higher in wet AMD patients than in controls. There were no significant differences in the BMI, MAP, iris color values or work conditions between the groups.

**TABLE 1:** Characteristics of the study groups.

**Parameter**	**Dry AMD group**	**Wet AMD group**	**Control group**	***p*-value^∗^**		
				**Dry AMD group vs. control group**	**Wet AMD group vs. control group**	**Dry AMD vs. wet AMD group**
Number of subjects	175	179	121	–	–	–
Sex (male/female)	52/123	83/96	32/89	0.60	**>0.001**	**0.001**
Patient’s age [years] (mean ± SD)	72.7 ± 8.0	74.14 ± 7.92	73.1 ± 6.0	1.00	0.33	0.16
BMI [kg/m^2^] (mean ± SD)	26.94 ± 4.29	26.93 ± 4.17	26.56 ± 3.66	1.00	1.00	1.00
WHR [arbitrary units] (mean ± SD)	0.88 ± 0.09	0.91 ± 0.09	0.88 ± 0.09	1.00	**0.02**	**0.006**
Waist circumference [cm] (mean ± SD)	91.52 ± 12.53	94.24 ± 12.65	90.13 ± 11.62	1.00	**0.04**	0.18
MAP [mmHg] (mean ± SD)	97.10 ± 10.40	99.50 ± 11.65	98.72 ± 9.66	1.00	1.00	0.29
Current smokers (%)	10.63%	16.56%	6.25%	0.27	**0.02**	0.14
Former smokers (%)	45.00%	57.67%	30.93%	**0**.**03**	**>0.001**	**0.03**
Smoking pack-years (mean ± SD)^	10.06 ± 16.08	17.04 ± 20.80	6.00 ± 13.09	0.12	**>0.001**	**0.01**
Period without smoking [years] (mean ± SD)	6.31 ± 10.78	7.26 ± 11.03	5.30 ± 10.23	0.92	**>0.001**	0.06
Iris color (dark/light)	45/129	46/132	26/95	0.41	0.41	1.00
Outdoor/indoor working conditions	38.29/61.71%	41.90/58.10%	33.06/66.94%	0.39	0.15	0.52
Hypertension (%)	65.00%	64.42%	71.13%	0.34	0.28	1.00
Duration of hypertension [years] (mean ± SD)	8.44 ± 9.56	7.92 ± 9.36	9.15 ± 9.86	0.45	0.22	0.64
History of ischemic heart disease (%)	15.09%	17.18%	11.34%	0.46	0.22	0.65
Duration of ischemic heart disease [years]	1.35 ± 4.75	1.11 ± 3.52	0.81 ± 3.28	0.42	0.21	0.59
(mean ± SD)						
History of cardiac infarction (%)	7.55%	4.91%	6.19%	0.80	0.78	0.36
History of cerebral stroke (%)	3.18%	2.45%	3.09%	1.00	0.71	0.75
History of peripheral artery disease (%)	3.77%	6.13%	6.19%	0.38	1.00	0.44
History of aortic aneurysm (%)	1.89%	1.25%	0.00%	0.29	0.53	0.68
Hypotensive drugs/vasodilators	65.00%	65.03%	70.10%	0.42	0.42	1.00
Hormonal drugs	20.00%	14.29%	20.62%	1.00	0.23	0.19
Thyroxine	15.63%	11.80%	20.62%	0.31	0.07	0.33
Steroids	2.50%	1.24%	1.03%	0.65	1.00	0.45
Other hormonal drugs	1.25%	1.24%	0.00%	0.53	0.53	1.00
Statins	26.88%	26.38%	36.08%	0.13	0.12	1.00
NSAIDs	17.50%	22.84%	19.59%	0.74	0.64	0.27
Cardiac medications/antiarrhythmic drugs	15.63%	12.27%	14.43%	0.86	0.70	0.42
Antiasthmatic drugs	5.63%	9.20%	3.09%	0.54	0.08	0.29
Antidepressants	5.00%	4.32%	5.15%	1.00	0.77	0.80

*In bold, *p*-value < 0.05, which was considered statistically significant. ^∗^Siegel and Castellan’s test/Fisher’s exact test; ^*p* < 0.05 for comparison between 3 groups, Kruskal–Wallis test; BMI, body mass index; WHR, waist-hip ratio; MAP, mean arterial pressure; NSAIDs, non-steroidal anti-inflammatory drugs.*

### Blood Count Analysis

First, we performed a complete blood count analysis in all enrolled subjects (Table 2). We found that the WBC count values and proportion of neutrophils were significantly higher, in the wet AMD group compared with the values in the control group, while the proportion of lymphocytes appeared to be lower in the wet AMD group compared with the values in the control group. Wet AMD appeared to be associated with a higher WBC (β = +0.19, *p* = 0.004), a higher proportion of neutrophils (β = +0.199, *p* = 0.003) and a lower proportion of lymphocytes (β = −0.17, *p* = 0.009) in the multivariate analysis performed using a GLM after adjustment for age, sex and smoking status of the patient (pack-years). There were no differences in blood count analysis between dry AMD and control group, however, multivariate analysis performed using a GLM after adjustment for age, sex and smoking status of the patient (pack-years) revealed that dry AMD was an independent factor associated with a higher proportion of neutrophils (β = +0.13, *p* = 0.05) and a lower proportion of lymphocytes (β = −0.14, *p* = 0.02).

**TABLE 2:** Complete blood count results in the study groups.

**Parameter**	**Dry AMD group**	**Wet AMD group**	**Control group**	**p-value^∗^**		
				**Dry AMD group vs. control group**	**Wet AMD group vs. control group**	**Dry AMD vs. wet AMD group**
WBC Count [^∗^10^3^/μl]^∧^	6.35 ± 1.83	6.89 ± 1.92	6.12 ± 1.71	0.91	**0.001**	**0.01**
Lymphocytes (%)^∧^	31.70 ± 8.33	30.64 ± 7.84	33.33 ± 8.79	0.35	**0.02**	0.58
Monocytes (%)	8.93 ± 1.98	8.67 ± 2.26	8.67 ± 1.78	1.00	1.00	0.48
Neutrophils (%)^∧^	55.98 ± 8.96	57.54 ± 9.08	54.70 ± 9.26	0.81	**0.02**	0.19
Eosinophils (%)	2.68 ± 1.70	2.51 ± 1.96	2.64 ± 2.28	1.00	1.00	0.22
Basophils (%)	0.71 ± 0.33	0.59 ± 0.36	0.66 ± 0.35	0.59	1.00	0.19
RBC Count [^∗^10^6^/μl]	4.62 ± 0.45	4.70 ± 0.38	4.62 ± 0.36	1.00	0.13	0.11
Hemoglobin [g/dl]	8.61 ± 0.82	8.75 ± 0.75	8.64 ± 0.62	1.00	0.34	0.18
Hematocrit [^∗^100%]^∧^	0.41 ± 0.04	0.42 ± 0.03	0.41 ± 0.03	1.00	**0.04**	0.07
MCV [fl]	89.00 ± 4.32	88.91 ± 4.35	88.64 ± 3.49	0.56	0.92	1.00
MCH [pg]	1.87 ± 0.11	1.86 ± 0.11	1.87 ± 0.08	1.00	1.00	1.00
MCHC [g/dl]	21.00 ± 0.63	20.97 ± 0.61	21.16 ± 0.57	0.19	0.07	1.00
RDW [%]^∧^	13.28 ± 1.17	13.53 ± 1.09	13.12 ± 0.91	0.94	**>0.001**	**0.008**
Platelets [^∗^10^3^/μl]	231.14 ± 61.17	225.03 ± 53.46	227.56 ± 59.77	1.00	1.00	1.00
MPV [fl]	10.49 ± 0.93	10.47 ± 0.87	10.43 ± 0.83	1.00	1.00	1.00

*In bold, *p*-values < 0.05, which were considered statistically significant. ^∗^Siegel and Castellan’s test; ^∧^*p* < 0.05 for comparison between 3 groups, Kruskal–Wallis test; WBC, white blood cells; RBC, red blood cells; MCV, mean corpuscular volume; MCH, mean corpuscular hemoglobin; MCHC, mean corpuscular hemoglobin concentration; RDW, red blood cell distribution width; MPV, mean platelet volume.*

### Inflammatory Factor Levels

To assess the systemic inflammatory response, we chose 11 factors for analysis in plasma: TNF-α, IL-1β, IL-2, IL-4, IL-5, IL-6, IL-8, IL-10, IL-12 p70, GM-CSF, and IFN-γ. The wet AMD group presented with higher levels of 3 (IL-6, GM-CSF, IFN-γ) and lower concentrations of 4 (IL-1β, IL-5, IL-10, IL-12) tested cytokines in comparison with the control group (Table 3). Multivariate analysis of patients and controls, adjusted for age, sex and smoking status (pack-years), revealed that wet AMD was an independent factor associated with higher concentrations of IL-6 (β = +0.24, *p* = 0.0004), GM-CSF (β = +0.31, *p* < 0.001) and IFN-γ (β = +0.58, *p* < 0.001) and lower concentration of IL-1β (β =−0.25, *p* = 0.0003), IL-5 (β = −0.45, *p* < 0.001), IL-10 (β = −0.45, *p* < 0.001) and IL-12 (β = −0.35, *p* < 0.001). Accordingly, dry AMD group presented with lower concentrations of 5 analyzed factors (IL-1β, IL-2, IL-5, IL-10, IL-12) and only one cytokine – GM-CSF concentration being higher as compared with controls. Multivariate analysis of patients and controls, adjusted for age, sex and smoking status (pack-years), revealed that dry AMD was an independent factor associated with lower concentrations of TNF-α (β = +0.24, *p* = 0.0004), IL-1β (β = −0.39, *p* < 0.001), IL-2 (β = −0.20, *p* = 0.003), IL-5 (β = −0.54, *p* < 0.001), IL-10 (β = −0.56, *p* < 0.001), IL-12 (β = −0.51, *p* < 0.001) and higher concentration of GM-CSF (β = +0.34, *p* < 0.001) and IL-6 (β = +0.13, *p* = 0.05). AMD subtype analysis revealed statistically significant differences in the concentrations of 7 tested factors (IL-2, IL-4, IL-5, IL-6, IL-10, IL-12, IFN-γ), all of which were elevated in the wet AMD group compared to the concentrations in the dry AMD group (Table 3). Interestingly, in AMD patients, we observed negative correlations between the percentage of lymphocytes and TNF-α (Rs = −0.14; *p* = 0.02), IL-2 (Rs = −0.14; *p* = 0.02), IL-5 (Rs = −0.17; *p* = 0.004), IL-6 (Rs = −0.19; *p* = 0.002), IL-10 (Rs = −0.18; *p* = 0.003), and IL-12 (Rs = −0.20; *p* = 0.001) and positive correlations between the percentage of neutrophils and TNF-α (Rs = +0.14; *p* = 0.02), IL-1β (Rs = +0.13; *p* = 0.03), IL-2 (Rs = + 0.15; *p* = 0.02), IL-5 (Rs = +0.16; *p* = 0.009), IL-6 (Rs = +0.18; *p* = 0.003), IL-10 (Rs = +0.18; *p* = 0.003), and IL-12 p70 (Rs = +0.21; *p* = 0.0008). Such correlations were not observed in the controls.

**TABLE 3:** Comparison of inflammatory factor levels in dry and wet AMD patients in comparison with control group.

	**Dry AMD group**		**Wet AMD group**		**Control group**		***p*-value^∗^**		
	***N***	**Mean ± SD**	***N***	**Mean ± SD**	***N***	**Mean ± SD**	**Dry AMD group vs. control group**	**Wet AMD group vs. control group**	**Dry AMD vs. wet AMD group**
TNF-α [pg/mL]	138	3.630 ± 2.019	135	3.908 ± 2.001	116	4.008 ± 1.702	0.09	1.00	0.59
IL-1β [pg/mL]^∧^	138	0.490 ± 0.264	135	0.571 ± 0.473	116	0.770 ± 0.572	**>0.001**	**>0.001**	0.27
IL-2 [pg/mL]^∧^	138	0.260 ± 0.551	135	0.415 ± 0.644	116	0.296 ± 0.325	**0.002**	1.00	**0.004**
IL-4 [pg/mL]^∧^	138	7.060 ± 7.197	135	10.613 ± 10.025	116	7.354 ± 5.810	0.60	0.33	**0.008**
IL-5 [pg/mL]^∧^	138	0.329 ± 0.289	135	0.438 ± 0.320	116	0.779 ± 0.531	**>0.001**	**>0.001**	**>0.001**
IL-6 [pg/mL]^∧^	138	2.899 ± 4.544	135	7.383 ± 31.517	116	1.802 ± 1.433	1.00	**0.02**	**0.04**
IL-8 [pg/mL]	138	4.701 ± 2.464	135	4.881 ± 2.946	116	4.427 ± 2.080	1.00	1.00	1.00
IL-10 [pg/mL]^∧^	138	0.737 ± 0.551	135	0.987 ± 1.224	116	1.696 ± 1.112	**>0.001**	**>0.001**	**0.009**
IL-12 p70 [pg/mL]^∧^	138	0.709 ± 1.257	134	1.112 ± 1.445	116	1.882 ± 1.448	**>0.001**	**>0.001**	**0.02**
GM-CSF [pg/mL]^∧^	138	0.427 ± 0.391	132	0.419 ± 0.516	116	0.271 ± 0.286	**>0.001**	**>0.001**	1.00
IFN-γ [pg/mL]^∧^	67	1.216 ± 0.656	70	2.496 ± 2.978	43	1.073 ± 1.616	1.00	**>0.001**	**>0.001**

*In bold, *p*-value < 0.05, which was considered statistically significant. N, number of observations; ^∗^Siegel and Castellan’s test; ^∧^*p* < 0.05 for comparison between 3 groups, Kruskal–Wallis test; TNF-α, tumor necrosis factor; IL-1β, interleukin-1β; IL-2, interleukin-2; IL-4, interleukin-4; IL-5, interleukin-5; IL-6, interleukin-6; IL-8, interleukin-8; IL-10, interleukin-10; IL-12 p70, interleukin-12p70; GM-CSF, granulocyte-macrophage colony-stimulating factor; IFN-γ, interferon-γ.*

Subsequently, we assessed the effect of the severity of underlying disease on the systemic inflammatory response and investigated the association between the concentrations of analyzed cytokines and cell counts and selected clinical parameters. Remarkably, we found negative correlations between visual acuity and selected analyzed inflammatory factors (Rs = −0.155, *p* = 0.01 for TNF-α; Rs = −0.13, *p* = 0.03 for IL-1β, Rs = −0.141, *p* = 0.02 for IL-2, Rs = −0.164, *p* = 0.006 for IL-4, Rs = −0.209, *p* = 0.0005 for IL-6, Rs = −0.147, *p* = 0.02 for IL-10), as well as negative correlations between visual acuity and WBC (Rs = −0.176, *p* = 0.0016) and lymphocyte count (Rs = −0.117, *p* = 0.036). Notably, no such correlations were found in the control group. Likewise, we observed significant associations between analyzed factors and retinal and choroidal parameters. We found negative correlations between central choroidal thickness values and selected analyzed inflammatory factors (Rs = −0.171, *p* = 0.005 for IL-5, Rs = −0.150, *p* = 0.01 for IL-10), as well as positive correlations between central retinal thickness values and WBC (Rs = +0.125, *p* = 0.023) and selected analyzed inflammatory factors (Rs = +0.163, *p* = 0.007 for IL-2, Rs = +0.143, *p* = 0.02 for IL-4, Rs = +0.292, *p* = 0.0005 for IFN-γ). Notably, no such correlations were observed in the control group. This indicates that patients with a more advanced stage of disease display higher plasma levels of inflammatory cytokines.

### Cellular miRNA Expression Profiles

Next, we performed a quantitative analysis of the expression of the selected miRNAs in peripheral blood cells (PBNCs) of AMD and control patients using qRT-PCR. We chose a panel of 13 miRNAs (miRNA-16-5p, -17-3p, -17-5p, -21-3p, -23a-3p, -30b, -126-3p, -126-5p, -146a, -150-5p, -155-5p, -191-5p, and -223-3p). Of the 13 analyzed miRNAs, four (miRNA-23a-3p, miRNA-30b, miRNA-191-5p, and miRNA-223-3p) showed higher expression, whereas four (miRNA-16-5p, miRNA-17-3p, miRNA-150-5p, and miRNA-155-5p) showed lower expression in the PBNCs of wet AMD patients compared with the expression in the PBNCs of control patients (Table 4). Multivariate analysis of patients and controls, adjusted for age, sex and smoking status (pack-years), revealed that wet AMD was an independent factor associated with higher expression of miRNA-23a-3p (β = +0.60, *p* < 0.0001), miRNA-30b (β = +0.32, *p* < 0.0001), miRNA-191-5p (β = +0.28, *p* < 0.0001) and lower expression of miRNA-16-5p (β = −0.31, *p* < 0.0001), miRNA-17-3p (β = −0.18, *p* = 0.01), miRNA-150-5p (β = −0.18, *p* = 0.01) and miRNA-155-5p (β = −0.47, *p* < 0.0001). Accordingly, dry AMD group presented with higher expression of four (miRNA-23a-3p, miRNA-126-3p, miRNA-126-5p, and miRNA 146a) and lower expression of three (miRNA-16-5p, miRNA-17-3p, and miRNA-17-5p) miRNAs in the PBNCs as compared with controls. Multivariate analysis of patients and controls, adjusted for age, sex and smoking status (pack-years), revealed that dry AMD was an independent factor associated with higher expression of miRNA-23a-3p (β = +0.60, *p* < 0.0001), miRNA-126-3p (β = +0.23, p = 0.0005), miRNA-126-5p (β = +0.16, *p* = 0.01) and miRNA 146a (β = +0.14, *p* = 0.03), mRNA191-5p (β = +0.15, *p* = 0.03) and lower expression of miRNA-16-5p (β = −0.23, *p* = 0.0004), miRNA-17-3p (β = −0.20, *p* = 0.003) and miRNA-17-5p (β = −0.19, *p* = 0.004). Cellular miRNA profiles were distinct between wet and dry AMD patients (Table 4). Of six differentially expressed miRNAs in AMD subtypes, four (miRNA-126-3p, miRNA-126-5p, miRNA-150-5p, and miRNA-155-5p) were upregulated in the PBNCs of dry AMD patients, whereas two (miRNA-30b and miRNA-191-5p) showed higher expression in the PBNCs of wet AMD patients.

**TABLE 4:** Cellular miRNA profiles in dry and wet AMD patients in comparison with control group.

	**Dry AMD group**		**Wet AMD group**		**Control group**		***p*-value^∗^**		
	***N***	**Mean ± SD**	***N***	**Mean ± SD**	***N***	**Mean ± SD**	**Dry AMD group vs. control group**	**Wet AMD group vs. control group**	**Dry AMD vs. wet AMD group**
miRNA-16-5p^∧^	153	0.726 ± 0.306	145	0.695 ± 0.175	112	0.802 ± 0.209	**>0.001**	**>0.001**	1.00
miRNA-17-3p^∧^	154	0.405 ± 0.604	144	0.481 ± 0.786	113	0.561 ± 0.838	**0.01**	**0.02**	1.00
miRNA-17-5p^∧^	154	0.942 ± 0.333	144	0.975 ± 0.786	112	1.056 ± 0.298	**0.003**	0.12	0.59
miRNA-21-3p	154	1.396 ± 0.995	145	1.188 ± 0.787	113	1.423 ± 2.626	0.55	1.00	0.29
miRNA-23a-3p^∧^	154	1.328 ± 0.573	145	1.257 ± 0.422	113	0.541 ± 0.517	**>0.001**	**>0.001**	1.00
miRNA-30b^∧^	154	0.810 ± 0.264	145	0.930 ± 0.328	112	0.749 ± 0.267	0.21	**>0.001**	**0.001**
miRNA-126-3p^∧^	154	0.449 ± 0.418	144	0.296 ± 0.255	113	0.280 ± 0.217	**0.002**	1.00	**>0.001**
miRNA-126-5p^∧^	154	0.302 ± 0.330	144	0.213 ± 0.230	112	0.220 ± 0.253	**0.02**	1.00	**0.006**
miRNA-146a^∧^	154	0.883 ± 0.477	145	0.795 ± 0.422	113	0.764 ± 0.253	**0.03**	0.83	0.32
miRNA-150-5p^∧^	154	1.605 ± 1.345	145	1.186 ± 1.066	113	1.400 ± 0.980	1.00	**0.04**	**>0.001**
miRNA-155-5p^∧^	154	0.507 ± 0.794	145	0.176 ± 0.537	112	0.542 ± 0.716	0.27	**>0.001**	**>0.001**
miRNA-191-5p^∧^	154	2.240 ± 1.286	145	2.509 ± 1.132	113	2.037 ± 1.165	0.78	**>0.001**	**0.02**
miRNA-223-3p	154	0.838 ± 0.420	145	0.842 ± 0.253	112	0.797 ± 0.350	0.80	**0.04**	0.11

*In bold, *p*-value < 0.05, which was considered statistically significant. N, number of observations; ^∗^Siegel and Castellan’s test; ^∧^*p* < 0.05 for comparison between 3 groups, Kruskal–Wallis test; Expression is calculated in relation to miR-93 as reference miRNA.*

Subsequently, we assessed the effect of the severity of underlying disease on the miRNA profile and investigated the association between the expression of analyzed molecules and selected clinical parameters. Remarkably, we observed positive correlations between visual acuity and selected miRNA expression (Rs = +0.243, *p* = 0.00002 for miRNA-126-3p; Rs = +0.199, *p* = 0.0005 for miRNA-126-5p; Rs = +0.144, *p* = 0.01 for miRNA-155-5p), as well as negative correlations between visual acuity and miRNA-191-5p (Rs = −0.153, *p* = 0.008). Notably, no such correlations were found in the control group. Likewise, we observed significant associations between the analyzed miRNA profile and retinal and choroidal parameters. We found a positive correlation between central choroidal thickness values and miRNA 126-3p expression (Rs = +0.149, *p* = 0.01), as well as significant correlations between the thickness of the central retina and the expression of miRNA-126-3p (Rs = -0.119, p = 0.04), miRNA-155-5p (Rs = −0.177, *p* = 0.002) and miRNA-191-5p (Rs = +0.175, *p* = 0.002). Notably, no such correlations were found in the control group.

### miRNA Correlations

To more accurately characterize the role of the analyzed miRNA profiles in AMD patients, we evaluated the association between the expression of miRNA molecules and inflammatory factor plasma level analysis. We aimed to investigate whether certain miRNAs are linked with inflammatory factors and whether these correlations are specific for AMD patients or wet/dry AMD subtypes. In general, we found that the expression of analyzed miRNA molecules strongly correlated with the levels of tested inflammatory factors in AMD patients, whereas such correlations were not observed in controls (Table 5). The miRNA with the highest expression from the tested panel in both dry and wet AMD groups, miRNA-191-5p, was significantly correlated with almost all inflammatory factor concentrations [TNF-α, IL-1β, IL-2, IL-4, IL-5, IL-6, IL-8, IL-12 (p70), and GM-CSF] in these subjects, while no such correlations were found in controls. Strong positive correlations between the expression of miRNA-191-5p and IL-2 and IL-6 levels in the wet AMD group corresponded to significant upregulation of both miRNA-191-5p, IL-2 and IL-6 in these patients. Similar positive correlations between this miRNA and these factors were also observed in dry AMD group, but not in controls. Negative correlations between two factors strongly elevated in wet AMD patients (IL-4, IL-6) and miRNA-30b and miRNA-146a were exclusive for this group and not observed in dry AMD or control group. In fact, these two miRNAs (miRNA-30b, miRNA-146a) showed only negative correlations (which were statistically significant) with most of the analyzed inflammatory factors in both dry and wet AMD groups. The observed diversity of miRNA-inflammatory factor correlations reflects the vast variety of biological processes that these miRNAs regulate.

**TABLE 5:** Spearman’s correlation coefficients for cellular miRNAs and inflammatory factors in dry and wet AMD patients in comparison with control group.

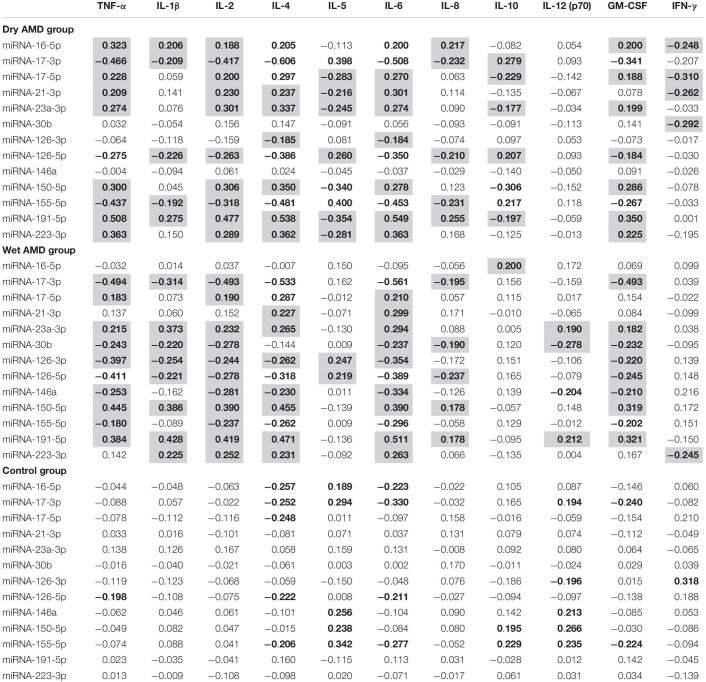

*Statistically significant results (p < 0.05) are shown in bold. The gray background corresponds to unique correlations, which were statistically significant only in AMD patients, not controls.*

## Discussion

AMD is a complex disease of the central retina and is a major cause of blindness in developed countries, significantly affecting the quality of life of millions. However, the pathogenesis of AMD is not currently completely understood. The involvement of various processes (e.g., oxidative stress, pathological angiogenesis, inflammation, and lipid oxidation) in AMD has been proposed, and it seems that both environmental and genetic factors are equally important in disease pathology. In this study, we focused on two potentially major contributors to AMD pathogenesis: inflammation and miRNAs. First, we aimed to address whether pathological processes in AMD, basically limited to the retina, could also be reflected in systemic changes in inflammatory factors and cellular miRNA expression profiles. We also attempted to correlate these results with selected clinical characteristics (e.g., visual acuity or central retinal/choroidal thickness) in AMD patients.

First, we performed an initial assessment of systemic markers of inflammation in AMD patients with a complete white blood cell count. The significantly increased WBC and neutrophil count and low lymphocyte number in wet AMD observed in this study are in concordance with previous research. In a large study of over 3,000 AMD patients, an elevated WBC count was linked with early AMD incidence, independent of smoking and other major confounders (Shankar et al., 2007). Recently, the neutrophil-to-lymphocyte ratio (NLR) and platelet-to-lymphocyte ratio (PLR) have been proposed as indicators of systemic inflammation; both ratios have been shown to be increased in AMD patients (Niazi et al., 2019). According to Sengul et al. (2017), chronic inflammation leads to increased neutrophil and monocyte counts and decreased lymphocyte counts due to augmentation of apoptosis. Although Pinna et al. (2018) recently suggested that blood count-derived inflammation biomarkers are unreliable in AMD, their study was performed in a small group of 79 male patients. The difference in NLR and PLR between dry and wet AMD subtypes also draws interest (Ilhan et al., 2015).

Next, we tested selected inflammatory factor concentrations in blood samples and found that many of them were dysregulated in AMD patients, specifically in the wet AMD subtype. In our study, the inflammatory factor profile in wet AMD patients was similar to that of previously published studies, especially with notable IL-2, IL-4, IL-5, IL-6, IL-10, IL-12, and IFN-γ increases (Seddon et al., 2005; Jonas et al., 2012). It was found that paracrine factors released from reactive retinal microglia can trigger NLRP3 inflammasome activation in the RPE cells, which in response augment expression of pro-inflammatory marker genes, such as IL-1ß, IL-6, IL-8, and GM-CSF in these cells (Nebel et al., 2017). The increased expression of pro-inflammatory cytokines including IL-1β and IL-6 in inflammasome-triggered RPE cells may indicate the more stressed RPE cells and highly negative effects on AMD progression *in vivo*. In this notion, in our study we found that wet AMD was associated with higher concentrations of several pro-inflammatory cytokines, such as IL-6, GM-CSF, and IFN-γ, whereas dry form of the condition is accompanying with higher levels of GM-CSF and IL-6. GM-CSF is generally recognized as an pro-inflammatory cytokine and its inflammatory activity is primarily due its role as a growth and differentiation factor for myeloid lineage cells including granulocyte and macrophage populations. Mostly, the pro-inflammatory effects of GM-CSF appear to depend on the dose and the presence of other relevant cytokines in the context of an immune response (Shachar and Karin, 2013). In the retina, GM-CSF promotes the survival and activation of microglia and perivascular macrophages (Hamilton, 2008). Importantly, Wang et al., found that GM-CSF is elevated in the vitreous humor of eyes having the at-risk homozygous CC variant in the complement factor H (CFH) gene (Wang et al., 2015). The polymorphism in this gene is a known genetic risk factor for AMD, where the CFH Y402H single nucleotide polymorphism is strongly associated with inflammation characterized by increased pro-inflammatory cytokines and oxidative stress-related molecules systemically in circulation and locally in the eye tissues (Wang et al., 2015). These data were also confirmed by the same group of Wang et al. (2015) through the *in vitro* studies, where they observed that stimulation of RPE cells with complement activation products, C3a and C5a, promotes upregulation of GM-CSF expression in RPE culture. In addition, stimulation of cultured RPE cells with proinflammatory TNF-α cytokine promoted strong secretion of GM-CSF to the environment *in vitro*. Moreover, GM-CSF may promote retinal neovascularization by releasing VEGF from neutrophils attracted to the subretinal space (Ohki et al., 2005). Besides, the correlations between AMD severity and WBC count and inflammatory factor levels observed in our study are worth noting. Negative correlations of pro-inflammatory cytokines with visual acuity shown in this study might bring us a step further in establishing the specific inflammation-related factor panel for potential use in diagnosis and monitoring of the progression of AMD.

To further explore the involvement of inflammation in AMD pathogenesis, we decided to study selected endogenous miRNA expression in peripheral blood circulating mononuclear cells, which are crucial components of complete immune system activity and thus may have important significance in modulating the inflammatory signaling pathways involved in the process of retinal degeneration in the course of AMD. Likewise, a recent report showed highly overexpressed endogenous miRNA-150 in circulating peripheral blood mononuclear cells from AMD patients, which could regulate macrophage-mediated inflammation and pathologic retinal angiogenesis independently from vascular endothelial growth factor (VEGF) (Lin et al., 2018). This finding was also correlated with an increased number of choroidal macrophages observed in human eyes with AMD, thus promoting pathological local neovascularization (McLeod et al., 2016). These data strongly implicate a pathogenic role of different intracellular miRNAs expressed in immune-related cells circulating in peripheral blood in AMD patients. Moreover, given that different miRNAs can act in a cell-specific and unique manner, potential differences in modulation of the inflammatory status by distinct immune-related cells highlight their importance in AMD pathogenesis and its progression. In our study, the PBNCs miRNA with the highest overall expression was miRNA-191-5p, and according to our knowledge, it has not yet been described in AMD patients. Importantly, miRNA-191-5p is a potent player in neuronal development (Kim et al., 2004) and differentiated malignant cell biology (Nagpal and Kulshreshtha, 2014), including retinoblastoma (McEvoy et al., 2012). In a recent study by Kumar et al. (2013) miRNA-191-5p was a part of the 7-miRNA signature, which could distinguish patients with Alzheimer’s disease (AD) from normal controls with great accuracy (AUC of 0.953). Since both AD and AMD share similarities in their intra- and extracellular deposits (Kaarniranta et al., 2011), the role of miRNA-191-5p in AMD could be significant. Specifically, Lykken and Li described the importance of miRNA-191-5p in supporting cytokine-dependent naïve, memory, and regulatory T-cell survival for homeostasis by controlling the levels of insulin receptor substrate-1 (IRS1) and thereby the activation kinetics of intracellular signaling with STAT5 (Lykken and Li, 2016). IRS1 associates with JAK1 and JAK3 in the absence of cytokines; IL-2 or IL-4 addition increases this association (Johnston et al., 1995). The positive correlation of miRNA-191-5p expression and IL-2 concentration in our study is in agreement with its biological function, similar to its correlation with IL-6, as IL-6 is an established target of miRNA-191-5p (Tufekci et al., 2010). Furthermore, the significant correlations of miRNA-191-5p expression with clinically important signs of retinal dysfunction, such as visual acuity (negative) and central retinal thickness (positive), might indicate a crucial role for this specific intracellular miRNA in AMD development and progression.

Another link between miRNA profiles in AD and AMD comes from a recent study on Aβ-injected rats and AMD patients, in which miR-9, miR-23, miR-2, miR-34, miR-146, and miR-155 were found to be dysregulated both in AMD and AD (Romano et al., 2017). A similar pattern of miRNA-23 and miRNA-146 upregulation and miRNA-155 downregulation, as we observed, in the aforementioned study was linked with several inflammatory signaling pathways (mTOR, TNFα, HIF signaling, and NF-κB) and insulin receptor signaling function, comparable to the mechanisms mentioned above for miRNA-191-5p. According to various studies, miRNA-155 exhibits both pro- and anti-inflammatory functions, depending on the stimulant involved (Rodriguez et al., 2007; Tahamtan et al., 2018). We found a negative correlation between miRNA-155 expression and inflammatory cytokine levels in AMD patients, which is in line with this miRNA pathway: it acts with IL-1 (Ceppi et al., 2009), TNF-α (Li et al., 2016), NF-κB (Wu et al., 2014), MyD88 and the inositol 5′-phosphatase SHIP-1 in infected macrophages (Bandyopadhyay et al., 2014). In miRNA-155-deficient mice, TNF-α and IL-6 are markedly increased (Yuan et al., 2016), while microglia and Treg lymphocyte cell numbers are reduced, possibly due to their impaired development (Kohlhaas et al., 2009; Yan et al., 2015). Furthermore, in a mouse model of oxygen-induced retinopathy, under ischemic conditions, downregulated miRNA-155 expression stimulated the increased inflammatory-related cytokine secretion and microglial activation, prompting aberrant angiogenic responses in the retina (Yan et al., 2015). Several other experiments performed *in vitro* and *in vivo* by other groups corroborated data obtained primarily in the miR-155 knock-out mice indicating that miR-155 may have a similar cell-type specific function in the microglia, endothelial cells, RPE cells and other cell types implicated in AMD pathogenesis (Berber et al., 2017). Thus, physiological miRNA-155 function appears to modulate inflammation-induced vascular injury and to induce its repair. In fact, we found miRNA-155 to be significantly downregulated in both examined forms of AMD compared to controls and to be even more substantially diminished in patients with the wet AMD type. In accordance with its inflammation-limiting role, the observed miRNA-155 positive correlation with visual acuity in our AMD group may further strongly highlight its role in linking inflammation and angiogenesis (Kutty et al., 2010; Zhuang et al., 2015).

miRNA-126 and miRNA-146, known as potent angiogenesis- and inflammation-regulating miRNAs, provide another important link between these processes in the pathogenesis of AMD (Guenther and Schrepfer, 2016). In our study, their endogenous expression was elevated in circulating cells of AMD patients and was correlated negatively with soluble inflammatory factors; miRNA-126 expression was also increased in dry AMD patients and correlated with clinical ophthalmological parameters. Both possess mostly anti-inflammatory roles, regulating NF-κB transcriptional activity and the biosynthesis of IL-1β, IL-6, IL-8, IL-10 and TNF-α and alleviating chemotactic effects toward macrophages via inhibition of TRAF6 activity (Kutty et al., 2013; Wu et al., 2017). miRNA-146 elevation has been widely studied in association with pro-inflammatory neurodegeneration in AD and AMD (Alexandrov et al., 2014). The upregulation of intracellular expression of both miRNA-126 strands (miR-126-3p and -5p) in immune-related cells in patients with dry AMD, together with their significant correlations with ophthalmological parameters related to retinal function, might suggest a general systemic attempt to suppress inflammation in remote organs or tissues, possibly similar to miRNA-146 overexpression, but the dual role of miRNA-126-3p and -5p strands in AMD angiogenesis remains to be further elucidated (Zhou et al., 2016).

Another two strands of intracellular miRNA that we studied were miRNA-17 (-3p, -5p), a component of the polycistronic miR-17-92 cluster, which is a negative regulator of angiogenesis. A previous study showed increased expression of extracellular miRNA-17 in the plasma of AMD patients (Ertekin et al., 2014) in contrast to our results observed intracellularly, although sample choice (plasma/cells) could have affected these discrepancies. MiRNA-17 is well known for its emerging role in inflammation (Kuo et al., 2019), in line with our results highlighting its correlation with inflammatory factor levels in the plasma of recruited AMD patients.

Altogether, the observed associations of the expression of different endogenous miRNAs in immune-related cells circulating in peripheral blood and several examined systemic inflammatory factors as well as differences between their expression in AMD/controls and wet/dry AMD groups may suggest the role of both intra- and extracellular mechanisms implicated in inflammatory response regulation in multifactorial AMD pathogenesis.

A limitation of the study is that the results cover one clinical time-point in the course of AMD. Moreover, this is an observational study and further experimental studies could be valuable. Particularly, experiments *in vivo* using miRNA knock-out mice could be considered to assess unique and overlapping biological characteristics of analyzed miRNAs and to unveil their pathological functions in these alive models. Such experimental studies could create a basis for potential therapeutic manipulation. Thus, we believe that the results described here form a solid basis for designing future studies to explore complex pathogenesis of AMD.

## Conclusion

In conclusion, we found significantly modified miRNA expression levels in peripheral blood cells (miRNA-16-5p, miRNA-17-3p, miRNA-17-5p, miRNA-23a-3p, miRNA-30b, miRNA-126-3p, miRNA-146a, miRNA-155-5p, and miRNA-191-5p) and soluble inflammatory factor concentrations in the plasma of AMD patients (IL-2, GM-CSF, IFN-γ, IL-1β, IL-5, IL-10, and IL-12), suggesting that, among others, these biologically active molecules have an important role in AMD. Our results could provide another tool to further explore the complex pathogenesis of this neurodegenerative disease, affecting elderly people. However, it remains to be determined whether these differences are a result of inflammatory processes in the retina in the course of AMD or if they are a reflection of a systemic response aimed at reducing the AMD-derived retinal damage or perhaps both. Further interventional studies with inflammation-modulating compounds might clarify to what degree the influence of inflammation is causative or contributory in AMD pathogenesis.

## Author Contributions

ZL wrote the manuscript. AS, KŁ, AG, and KM-P conducted the experiment. KM-P provided the materials. KS, MK, and AM analyzed and interpreted the data. MK, BM, and AM proofed and revised the manuscript. AM designed the experiment.

## Conflict of Interest

The authors declare that the research was conducted in the absence of any commercial or financial relationships that could be construed as a potential conflict of interest.
